# Supplementary material for: Splice-Junction-Based Mapping of Alternative Isoforms in the Human Proteome
Source: Cell Rep. Author manuscript; Available in PMC 2020 Jan 15. (PMC6961840; doi:10.1016/j.celrep.2019.11.026)
Supplement: 2 [file NIHMS1546469-supplement-2.zip › DF1/PXD009737/TestisLysC/TestisLysC_20_PTGDS_GPGEDFRMATLYNK.pdf]

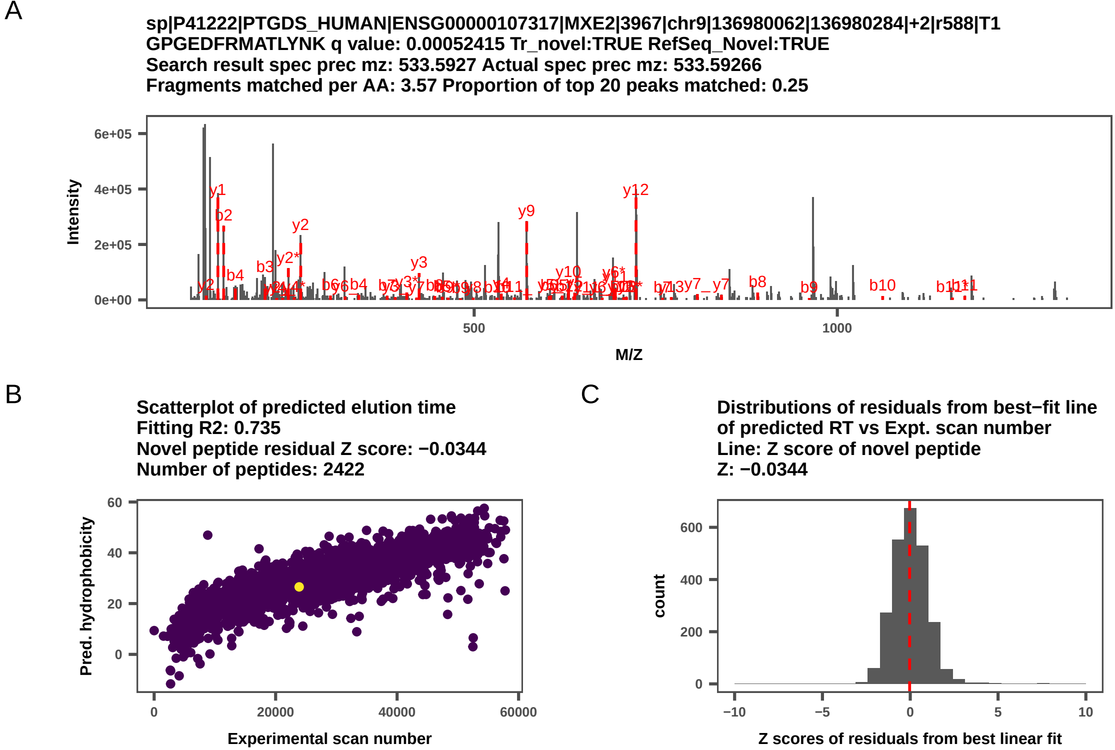

A
sp|P41222|PTGDS_HUMAN|ENSG00000107317|MXE2|3967|chr9|136980062|136980284|+2|r588|T1
GPGEDFRMATLYNK q value: 0.00052415 Tr_novel:TRUE RefSeq_Novel:TRUE
Search result spec prec mz: 533.5927 Actual spec prec mz: 533.59266
Fragments matched per AA: 3.57 Proportion of top 20 peaks matched: 0.25
Intensity
6e+05
4e+05
2e+05
0e+00
y1
b2
y2
b4
b3
y2*
y3
y9
y12
y10
y6*
b8
b9
b10
500
1000
M/Z
B
Scatterplot of predicted elution time
Fitting R2: 0.735
Novel peptide residual Z score: −0.0344
Number of peptides: 2422
Pred. hydrophobicity
60
40
20
0
0
20000
40000
60000
Experimental scan number
C
Distributions of residuals from best−fit line
of predicted RT vs Expt. scan number
Line: Z score of novel peptide
Z: −0.0344
count
600
400
200
0
−10
−5
0
5
10
Z scores of residuals from best linear fit
